# Nurses’ attitudes and knowledge about organ donation and transplantation in closed hospital wards

**DOI:** 10.2478/jccm-2025-0041

**Published:** 2025-10-31

**Authors:** Anastasios Tzenalis, Elpida Kontesidou, George Kipourgos, Evangelia Andreopoulou, Angelikh Gkotsi, Eleni Albani

**Affiliations:** University of Patras, Patra, Greece; General Hospital Papageorgiou, Thessaloniki, Greece; University General Hospital of Patras, Patra, Greece

**Keywords:** organ donation, nurses’ knowledge, transplants, brain death

## Abstract

**Objective:**

Nurses in closed hospital wards, such as Intensive Care and isolation units, play a pivotal role in identifying potential donors and supporting families during sensitive decision-making moments. However, gaps in knowledge or negative attitudes among nurses can hinder donation efforts. This study aims to explore the knowledge and attitudes of closed-ward nurses regarding organ donation and transplantation, providing insights to enhance education, advocacy, and clinical practices in these critical settings.

**Methods:**

Modern analysis was performed on the data collected from questionnaires distributed to nurses of … General Hospital. The study involved 108 nurses. The questionnaire used to collect the data was provided by the Department of Social Work of the … University and distributed in electronic form to hospital nurses.

**Results:**

After analyzing the nurses’ responses, it emerged that 85.19% of nurses are positive about the idea of organ donation and declare themselves willing to become donors, motivated by their will to really help their fellow human beings. In contrast, 5.56% said they would not be willing to donate. The main cause of their refusal seems to be fear and the prejudices they have, but also the fact that there is no trust in the organizations responsible for transplants. Finally, regarding the knowledge of the nurses who participated in the survey, the average knowledge score on the scale 0–100 is 72.

**Conclusions:**

There is a clear need for specialized training for nurses managing organ donation. The emotional burden and responsibilities they face are significant. Enhanced training supports their well-being and ensures a more compassionate, efficient process for donors and families, ultimately improving the experience for all involved.

## Introduction

Organ donation and transplantation are globally recognized as transformative medical interventions that save countless lives [1]. Despite advancements in medical technology, the success of these programs depends on two critical factors: public awareness and the active involvement of healthcare professionals, particularly nurses [2].

Organ donation is a life-saving option for patients with end-stage organ failure, yet donation rates often remain suboptimal due to lack of awareness, cultural barriers, and insufficient support mechanisms. In this context, healthcare professionals—and especially nurses—play a decisive role in bridging these gaps.

ICU nurses, in particular, are uniquely positioned to identify potential organ donors, provide emotional support to families, and coordinate with transplant teams. Their clinical insight and communication skills directly influence the success of the donation process. Nurses are often the first point of contact with potential donors and their families, and they must navigate emotionally charged and ethically complex situations, especially in closed hospital wards such as intensive care units (ICUs) and psychiatric or isolation wards [3].

Existing research highlights the significant impact of nurses’ knowledge and attitudes on the organ donation process [4]. Adequate knowledge enables nurses to provide accurate education and guidance to families, while positive attitudes enhance their capacity to advocate for organ donation [5]. Conversely, deficiencies in knowledge or negative perceptions can hinder the process, perpetuate misconceptions, and contribute to ethical dilemmas [6]. For example, limited understanding of brain death criteria or organ retrieval protocols may lead to delays or obstacles in the donation process, undermining its efficacy [7].

Closed hospital wards present a unique context for exploring these dynamics. Nurses in these settings are frequently exposed to critically ill or high-risk patients, where the potential for organ donation is heightened [8]. However, challenges such as high-pressure environments, inadequate training, and cultural or religious misconceptions can impede nurses’ ability to address organ donation effectively [9]. Emotional discomfort in communicating with families during end-of-life care further compounds these barriers, emphasizing the need for targeted interventions [10].

This study aims to evaluate the knowledge, attitudes, and perceptions of nurses working in closed hospital wards regarding organ donation and transplantation. By identifying gaps in understanding and barriers to effective practice, the research seeks to inform the development of tailored educational programs and enhance clinical protocols. These outcomes have broader implications for strengthening the organ donation framework, ensuring equitable access to life-saving transplants, and addressing the unique ethical and clinical challenges posed in closed-ward settings.

## Methods

### Study Design

This study was conducted among nurses working at Papageorgiou Hospital in Thessaloniki, Greece. A total of 108 nurses participated in the study, providing responses to a structured questionnaire distributed electronically. The study was designed to assess their knowledge, experiences, attitudes, and intentions regarding organ donation.

### Ethical Approval

Ethical approval for the study was obtained from the Board of Directors of Papageorgiou General Hospital, following a thorough review of the research protocol (Protocol Number: 35427/21-12-2023) Participation was voluntary, and written informed consent was collected from all participants prior to their involvement. Confidentiality and anonymity were ensured throughout the study in accordance with ethical standards.

### Participants

Nurses employed at Papageorgiou Hospital were eligible to participate. Data collection took place over a four-month period, from November 2023 to February 2024. Of the 150 questionnaires distributed electronically, 108 were completed, yielding a response rate of 72%.

### Data Collection

The survey instrument used for data collection was a validated questionnaire developed in 2008 by Associate Professor Ioannis Chliaoutakis from the Department of Social Work at the Hellenic Mediterranean University. The questionnaire, which has been extensively employed in prior research, was specifically designed to evaluate healthcare professionals’ knowledge, experiences, and attitudes regarding organ donation.

The questionnaire consisted of four sections. The first section gathered demographic information, including participants’ age, gender, educational background, years of professional experience, and work-place characteristics. The second section assessed participants’ knowledge of organ donation through 15 correct/ wrong questions, which covered key aspects such as the principles of organ donation, clinical processes, and eligibility criteria. The third section explored individual experiences with organ donation, incorporating five targeted questions aimed at capturing individual or professional encounters related to the topic. The last section investigated attitudes and intentions toward organ donation, comprising 16 structured items that evaluated participants’ perspectives, beliefs, and willingness to engage with organ donation practices.

### Statistical Analysis

Data were collected via Google Forms platform and analyzed using IBM SPSS Statistics (v.28). The Kolmogorov-Smirnov test was applied to assess the normality of quantitative variables. Associations between a quantitative variable and a dichotomous qualitative variable were analyzed using the Student’s t-test. For categorical variables with more than two levels, the ANOVA test was used where appropriate. Pearson’s correlation was applied exclusively for correlations between quantitative variables.

## Results

### Demographic Characteristics

A total of 108 nurses participated in the study. The majority were female (83.33%), while males accounted for 16.67%. Regarding age distribution, 14.81% were 20–29 years old, 20.37% were 30–39 years old, 50% were 40–49 years old, and 14.81% were 50–59 years old.

Participants were employed in various specialties: 33.33% in anesthesiology, 24.07% in general surgery, and 40.74% in intensive care units (ICUs). A small percentage (1.85%) worked in specialized ICUs. The mean professional experience was 15.51 years (SD=9.2 years).

In terms of professional roles, 70.37% were clinical nurses, 14.81% were head nurses, 12.96% were educators, and 1.85% held administrative positions. Regarding educational background, 37.04% had a master’s degree, 37.04% held a technological diploma, 18.52% had secondary education (nurse assistants), and 7.41% had a university degree. Detailed demographic characteristics are presented in Table I .

**Table I.** Demographic characteristics of nurses

**Characteristics of Nurses**	**Ν(%)**
Gender	
Male	18 (16,67)
Female	90 (83,33)

Age	
20–29	16 (14,81)
30–39	22 (20,37)
40–49	54 (50)
50–59	16 (14,81)

Department	
Anesthesiology	36 (33,33)
General surgeries	26 (24,07)
General ICU	44 (40,74)
SCICU	2 (1,85)

Workplace	
Clinical Instructor	14 (12,96)
Clinical nurse	76 (70,37)
Head Nurse	2 (1,85)
Charge Nurse	16 (14,81)

Level of training	
Master’s degree	40 (37,04)
Secondary degree	20 (18,52)
University degree	8 (7,41)
Technical degree	40 (37,04)

Participation as a conference presenter	
Yes	32 (29,63)
No	76 (70,37)

Attended a conference in the past year	
Yes	64 (59,26)
No	44 (40,74)

### Level of Knowledge

Participants exhibited varying levels of knowledge regarding organ donation. The mean knowledge score was 72.62% (SD=13.65%). Detailed responses to knowledge-related questions are provided in Table II.

**Table II:** Knowledge about organ donation

	**Correct answer**	**Wrong answer**	**I do not know**
The church allows organ donation.	44 (40,74)	44 (40,74)	20 (18,52)
You have to be perfectly healthy to be a donor.	38 (35,19)	56 (51,85)	14 (12,96)
There is a waiting list for potential recipients.	104 (96,3)	2 (1,85)	2 (1,85)
Histocompatibility between donor and recipient is decisive for the selection of the second.	100 (92,59)	6 (5,56)	2 (1,85)
In the case of brain death, relatives decide whether to donate an organ regardless of what the patient has declared while was alive.	54 (50)	40 (37,04)	14 (12,96)
There is a relevant legal framework for organ donation and transplantation.	96 (88,89)	0 (0)	12 (11,11)
In order to remove an organ from a potential donor, the donor must be brain dead.	102 (94,44)	0 (0)	6 (5,56)
The brain dead donor is tested for communicable diseases.	94 (87,04)	2 (1,85)	12 (11,11)
I can change my mind while I am a registered donor.	78 (72,22)	2 (1,85)	28 (25,93)
You can become an organ donor regardless of your age.	26 (24,07)	54 (50)	28 (25,93)
Brain death and coma are identical concepts.	92 (85,19)	12 (11,11)	4 (3,7)
Brain death is reversible.	100 (92,59)	2 (1,85)	6 (5,56)
In the case of brain death, the donor may have predetermined where the organs will be given.	86 (79,63)	4 (3,7)	18 (16,67)
Organ donation saves lives.	108 (100)	0 (0)	0 (0)

40.74% correctly identified the church’s supportive stance on organ donation, whereas an equal percentage believed otherwise.

Concerning the misconception that donors must be in perfect health, 35.19% correctly answered “no,” while 51.85% incorrectly assumed otherwise.

96.3% were aware of the existence of a transplant waiting list, and 92.59% understood the necessity of donor-recipient compatibility.

50% correctly recognized that the family makes the final donation decision after brain death, whereas 37.04% disagreed.

88.89% were knowledgeable about the legal framework surrounding organ donation, and 94.44% correctly stated that organs can only be harvested from brain-dead donors.

The distinction between brain death and coma was understood by 85.19% of participants, while 92.59% recognized the irreversibility of brain death.

All participants (100%) acknowledged that organ donation saves lives.

### Variable Correlations

Statistical analysis revealed that nurses’ knowledge levels were not significantly associated with gender (p=0.081), department of employment (p=0.098), years of experience (p=0.067), or job position (p=0.102). However, age was negatively correlated with knowledge scores (p=0.032, r=−0.256), indicating that older nurses demonstrated lower knowledge levels.

Furthermore, educational attainment did not significantly correlate with knowledge levels (p=0.063), nor did prior participation in seminars (p>0.08). However, having a close acquaintance who wished to be an organ donor was significantly associated with higher knowledge scores (p<0.046).

### Experience and Sources of Information

A total of 87.04% of participants reported that no family member had required an organ transplant, while 11.11% had such an experience. These findings are in Table III.

**Table III:** Experience of organ donation

	**Yes**	**No**	**I do not know**
Has a relative or friend ever needed a transplant?	12 (11,11)	94 (87,04)	2 (1,85)
Has any relative or friend of yours formally stated that they would like to become an organ donor?	40 (37,04)	50 (46,30)	18 (16,67)
Is any relative or friend interested in becoming a donor?	38 (35,19)	28 (25,93)	42 (38,89)
Does your environment have a negative attitude towards organ donation?	10 (9,26)	76 (70,37)	22 (20,37)
Would you ever become an organ donor?	92 (85,19)	10 (5,56)	5 (5,56)

The primary sources of information were the medical environment (42.59%), conferences and events (29.62%), and mass media (28.70%).

### Intention to Donate

A considerable proportion (85.19%) of participants expressed willingness to become organ donors, while 5.56% were opposed. The primary motivation for donation was altruism (95.64%). These findings are detailed in Table IV.

**Table IV.** Intention to donate

**I would become a donor because:**	**I strongly disagree**	**I disagree**	**I agree**	**I strongly agree**	**I do not know**
I truly want to help my fellow human beings.	2 (2,17)	2 (2,17)	24 (26,08)	64 (69,56)	0 (0)
By donating an organ, you save a life, which aligns with my religious beliefs.	4 (4,34)	4 (4,34)	28 (30,43)	54 (58,69)	2 (2,17)
I have been influenced by a relative or friend who is also a donor.	20 (21,73)	50 (54,34)	20 (21,73)	0 (0)	2 (2,17)
I have been sensitized by a relative or friend who needed a transplant.	24 (26,08)	44 (47,82)	18 (19,56)	4 (4,34)	2 (2,17)
I would only become a donor for someone close to me	32 (34,78)	40 (43,47)	12 (13,04)	4 (4,34)	4 (4,34)
The media has influenced me in favor of becoming a donor.	12 (12,04)	40 (43,47)	26 (28,26)	14 (15,21)	0 (0)
The title of being a donor gives me satisfaction and pride	2 (2,17)	22 (23,91)	44 (47,82)	20 (21,73)	4 (4,34)
By becoming a donor, I know that one of my organs will live on after my death	8 (8,69)	22 (23,91)	34 (36,35)	22 (23,91)	6 (6,52)

Age (p=0.65), department of employment (p=0.09), and years of service (p=0.32) did not significantly influence donation intent. However, female participants exhibited a higher willingness to donate compared to males (p=0.034), though the small number of male respondents may have affected this result.

Additionally, nurses with higher educational attainment were significantly more likely to express a positive attitude toward organ donation (p=0.008).

### Factors of Hesitation

Among the 10 participants who expressed concerns about organ donation, several key themes emerged. The primary reasons included fear (reported by 60%), distrust in transplant organizations (70%), and concerns about whether organs are used appropriately (80%). Notably, all ten nurses (100%) stated that they lacked comprehensive information about the donation process. Half of them (50%) also expressed doubts regarding the definition of brain death, indicating a need for greater clarity and education on this concept.

These findings suggest that enhancing education and fostering trust in medical institutions could significantly improve acceptance of organ donation among nurses.

## Discussions

### Factors that influence nurses’ knowledge regarding transplantation

The findings from the study highlight several critical factors that influence nurses’ knowledge regarding organ donation and transplantation. While the majority of participants demonstrated a solid understanding of core concepts, such as the irreversibility of brain death and the existence of legal frameworks for transplantation, significant gaps and misconceptions persist in specific areas.

It is worth noting that ICU nurses, due to their front-line role in managing critically ill patients, demonstrated slightly higher knowledge scores. Although not statistically significant, this trend reflects their frequent exposure to brain death diagnoses and transplant coordination, reinforcing the need for ongoing targeted training.

One notable gap is the widespread misconception that organ donors must be “perfectly healthy” or that age is a limiting factor for donation [11]. This indicates a lack of awareness about the inclusivity of organ donation criteria, which allows individuals of varying health statuses and ages to contribute, as demonstrated by our study results. Such misunderstandings may stem from insufficient exposure to updated medical guidelines or a lack of targeted training on organ donation protocols [12]. This is further supported by the finding that 35.19% of nurses incorrectly assumed that donors must be in perfect health. This suggests a pressing need for more comprehensive educational programs tailored specifically to healthcare professionals, particularly nurses, to ensure that they can accurately disseminate information to the public and patients [13]. Several studies show that a negative decision by the family is traced to the inability of health care professionals to communicate empathetically and sensitively and to a lack of trust in the care that is delivered to family members [14,15].

Legal and ethical considerations also emerged as areas where knowledge was strong but not universal. Most participants correctly understood the legal frameworks governing transplantation and the concept of brain death, yet a small proportion remained uncertain. For example, 85.19% of participants correctly identified the distinction between brain death and coma, while 92.59% acknowledged its irreversibility, but uncertainty still persisted among the remaining participants. This highlights the potential for reinforcing this knowledge through ongoing professional development and workshops, particularly as legal and ethical standards evolve over time [16]. Nurses who are confident in their understanding of these aspects are better equipped to advocate for organ donation and address patients’ or families’ concerns effectively [17].

Misinformation and uncertainty about medical criteria for donation, such as the requirement for brain death or the process for testing donors, also reveal systemic gaps in education. Despite relatively high levels of correct responses, the presence of uncertainty among even a small percentage of participants (e.g., 5.56% were unsure about brain death) is concerning, given nurses’ pivotal role in guiding decisions about organ donation [18]. This calls for standardized training across health-care institutions, emphasizing both the technical and procedural aspects of transplantation [19].

### Factors that influence nurses’ attitudes regarding transplantation

This study also highlights several factors that influence nurses’ attitudes toward organ donation and transplantation, shedding light on the complexities surrounding their reluctance to participate as donors. The findings suggest that the nurses’ attitudes are shaped by a combination of emotional, educational, and systemic factors, underscoring the need for targeted interventions to address these barriers.

Fear emerged as a significant factor influencing reluctance toward organ donation. The results show that 60% of those reluctant to donate cited fear as a reason. This indicates that emotional responses, potentially driven by misconceptions about the donation process, remain a substantial obstacle. Fear could stem from concerns about the physical implications of donation or the psychological unease associated with the thought of death and organ removal [20]. These findings align with previous studies that have identified fear as a recurring theme in the general population’s reluctance to donate organs [21,22], recognizing the need for interventions that address these emotional barriers through counseling and awareness programs.

Like in the study of Damar, Ordin, & Top, (2019) distrust in organizations and healthcare systems emerged as a factor influencing nurses’ reluctance to become organ donors [23]. This is supported by the study findings, where 70% of reluctant participants expressed distrust in transplant organizations. These findings emphasize the importance of fostering transparency and accountability within healthcare organizations to build trust among healthcare workers.

The total number of nurses who were reluctant to become donors agreed or strongly agreed that they were not fully informed or settled on the topic of organ donation. This unanimous finding highlights a critical educational gap among nurses, who are often perceived as key advocates for organ donation. The Skowronsk et al., (2021) also recognized the need for more and continuous education on organ donation [24], and the Pelicic et al (2019) noted that there is a lack of guidelines to support donation [25]. Moreover, 50% of the participants in our research expressed doubts about the concept of brain death, agreeing that hope for life exists until the last moment. These doubts further underscore the need for targeted educational initiatives to address misconceptions about brain death and the scientific basis of organ transplantation. This is confirmed by the Umana et al. (2018) study, which emphasized the need to raise awareness by implementing educational programs among health professionals on organ donation and transplantation [26]. Increasing knowledge has been consistently linked to more positive attitudes toward organ donation, making this an essential area for intervention [27].

Indifference did not appear to be a significant factor influencing nurses’ decision to become donors. However, lack of active engagement with the issue may contribute to hesitation, even among health professionals. Encouraging discussions about organ donation within professional and personal contexts could help address this passive reluctance [28].

From nurse’s answers appears that the concerns about brain death and the belief that hope persists until the last moment reflect deeper cultural or ethical considerations. For instance, 42.5% of respondents were not clear about their attitude toward donation. Religious beliefs, as a guiding framework of values, significantly influence decisions regarding organ donation [29]. Studies have shown that religious concerns rank among the most impactful factors leading to the refusal of organ donation [30]. Can and Hovardaoglu (2017) emphasized that concepts such as brain death and bodily integrity can conflict with religious principles, creating moral dilemmas [14], a conclusion also supported by others research [29,30]. Nurses, as caregivers, may struggle with reconciling the finality of brain death with their role in providing hope and care for patients. These attitudes could be influenced by societal norms, religious beliefs, or personal values [31], all of which warrant further exploration in future research. Türkiye is a country with a significant Islamic religious influence which impacts the attitude towards organ donation, a recent study in Türkiye, showed that the health personnel have a higher negative attitude and 42.5% of respondents were not clear about their attitude towards donation [23]. These findings suggest that engaging religious leaders in promoting organ donation could effectively bridge the gap between religious values and organ donation advocacy.

## Conclusion

This study highlights the strong altruistic motivation among nurses regarding organ donation. Despite this, persistent misconceptions and emotional barriers remain. Efforts should focus on targeted education, emotional support, and professional development to empower nurses in promoting organ donation.
